# Machine learning in scientific grant review: algorithmically predicting project efficiency in high energy physics

**DOI:** 10.1007/s13194-022-00478-6

**Published:** 2022-07-23

**Authors:** Vlasta Sikimić, Sandro Radovanović

**Affiliations:** 1 Cluster of Excellence – Machine Learning for Science and the Hector Research Institute of Education Sciences and Psychology, University of Tübingen, Tübingen, Germany; 2 Faculty of Organizational Sciences, University of Belgrade, Belgrade, Serbia

**Keywords:** Peer-review, Predictive analysis, Epistemic utility, Efficiency of experiments, Data envelopment analysis, High energy physics

## Abstract

As more objections have been raised against grant peer-review for being costly and time-consuming, the legitimate question arises whether machine learning algorithms could help assess the epistemic efficiency of the proposed projects. As a case study, we investigated whether project efficiency in high energy physics (HEP) can be algorithmically predicted based on the data from the proposal. To analyze the potential of algorithmic prediction in HEP, we conducted a study on data about the structure (project duration, team number, and team size) and outcomes (citations per paper) of HEP experiments with the goal of predicting their efficiency. In the first step, we assessed the project efficiency using Data Envelopment Analysis (DEA) of 67 experiments conducted in the HEP laboratory Fermilab. In the second step, we employed predictive algorithms to detect which team structures maximize the epistemic performance of an expert group. For this purpose, we used the efficiency scores obtained by DEA and applied predictive algorithms – lasso and ridge linear regression, neural network, and gradient boosted trees – on them. The results of the predictive analyses show moderately high accuracy (mean absolute error equal to 0.123), indicating that they can be beneficial as one of the steps in grant review. Still, their applicability in practice should be approached with caution. Some of the limitations of the algorithmic approach are the unreliability of citation patterns, unobservable variables that influence scientific success, and the potential predictability of the model.

## Introduction

Grant peer-review has been extensively criticized as time-consuming (Herbert et al., 2013), subjective (Guthrie et al., 2019), and costly as it requires scientists to spend time on the evaluation of project proposals than on research. On the other hand, machine learning has already been used for prescreening job applications in industry (e.g., Peñalvo et al., 2018) and for finding referees for grant peer-review in academia (Cyranoski, 2019). Furthermore, there is a discussion about the use of algorithms to identify the subjects of largest importance for speeding up the Decadal Survey on Astronomy and Astrophysics (Woodall, 2021). This survey is used for prioritizing research funding in areas such as exoplanets by funding agencies and the United States Congress (Woodall, 2021). The question is whether predictive algorithms could play a direct role in the process of evaluating academic projects, e.g., as a prescreening method.

In order to investigate the potential of machine learning in grant review and project evaluation, we analyzed the efficiency of experiments in HEP. Our goal was to algorithmically predict the project’s success based on the data from the funding proposal as a means of machine learning based grant review. For this purpose, we propose a two-stage method, which represents a novel approach to the optimization of scientific projects. The predictive analysis should discern the epistemic efficiency of an experiment, based on relevant data for each experiment. The data we used were numerically expressible and publicly available resources requested in project proposals. However, the proposed method is flexible and allows for the use of different, potentially larger, data sets.

In the context of our study, epistemic efficiency is technically defined as achieving the maximal outputs given all the inputs. The epistemic efficiency of experiments can also be understood in broader terms as an optimum reached by minimizing epistemic efforts while maximizing epistemic gain.

Previously, several attempts for evaluating project efficiency in HEP based on numerically expressed data have been made. For example, in a three-part assessment (Irvine & Martin, 1984; Martin & Irvine, 1984a, b) the research output of CERN was compared with other HEP laboratories and the contributions of individual accelerators within CERN were evaluated. The number of published papers and the number of citations were the core of this metric.

The repository INSPIRE-HEP (https://inspirehep.net/) offers extensive data about experiments conducted in the HEP laboratories CERN, DESY, Fermilab, and SLAC. Specifically, the INSPIRE database of Fermilab experiments consists of the following datasets: the number of papers within the citation groups (excluding self-citations), the number of proposing authors, the number of teams, and the exact duration of the experiment.^1^ These data are suitable for data mining computational analysis and other types of machine learning techniques. For instance, Perović et al. (2016) employed a DEA on data from Fermilab and demonstrated that the smaller projects outperform the larger ones in terms of efficiency scores. More specifically, smaller projects had better outcomes (in terms of the number of papers within the citation groups) given the inputs (in terms of the number of proposing authors, the number of teams, and the exact duration of the experiment). In the present paper, we go one step further and analyze whether one could use the quantitative data from the project proposals to predict their efficiency and, thus, contribute to the grant review in HEP.

Our twofold method for the estimation and prediction of the efficiency of HEP experiments consists of the following. In the first phase, we estimate efficiency, while in the second phase a model for the prediction of efficiency is provided. The method is flexible, meaning that any model can be used both for efficiency estimation and for efficiency prediction. In our research, we conducted a DEA on the data from 67 experiments that were run in the middle stage of the development of the HEP laboratory Fermilab.^2^ As inputs, we used the information present in the project proposals such as the duration of the project, the number of research teams, and the number of researchers. The outputs of the projects were the numbers of papers grouped by citation count into six categories. In the second phase, we conducted the predictive analysis of efficiency scores obtained in the previous step using several algorithms. Finally, we used heatmaps to visualize how efficiencies differ depending on the input values.

The increased use of machine learning algorithms in all spheres of our lives requires an analysis of whether they are applied responsibly. Studies about their applications on a new problem cannot predict all possible problems that could arise after their implementation, but can significantly anticipate some limitations of the approach. Our paper is focused on the question of whether machine learning algorithms can be used in grant review, under which limitations they should be used, and how to implement them responsibly.

The limitations that we anticipate and analyze are the following. First, the proposed metrics rely on citations which are not always an adequate indicator of the research success (e.g., Contopoulos-Ioannidis et al., 2008; Fister Jr et al., 2016). Secondly, many unobservable variables, such as the motivation of team members and their cohesion, play an important role in the project outcome. Thirdly, if the grant review were exclusively based on machine learning algorithms, the possibility of the gamification of the grant-writing would increase. More precisely, applicants could start adjusting their proposals based on the parameters from the previous successful projects to defeat the algorithms. Thus, we argue that one should use machine learning with caution and ideally combine it with peer-reviews.

The application of algorithmic techniques for the evaluation of scientific projects is relevant for the data analysis community because it represents the use of their techniques in a new domain. Also, from the standpoint of social epistemology of science, one is in a position to make precise instructions about which team structure maximizes the knowledge acquisition within a group. Moreover, this research is important for science policy as well as scientists working on these projects, because it helps to make practical decisions about the project structure. Based on all the results one should be in the position to decide whether a specific project should be funded, how to structure a project, and finally which are the tendencies in project optimization. Thus, the main contributions of the paper are:

- a methodology for the estimation and prediction of the efficiency of HEP experiments;
- efficiency estimation of 67 experiments in HEP;
- results of a predictive analysis and evaluation of its potentials and limitations.

The paper is organized in the following way. The motivation for studying the efficiency of knowledge acquisition, i.e., epistemic efficiency of scientific projects is discussed in Section 2. In Section 3 we propose and discuss in detail a twofold method for predicting efficiency in HEP, while in Section 4 we present the results of our study on data from Fermilab and analyze them. In Section 5, we discuss the limitations and potentials of algorithmic grant peer-review. Finally, in Section 6 we conclude the paper by pointing out the potential of data-driven analyses for the optimization of research beyond experimental physics.

## Motivation: Efficiency in producing scientific knowledge

Research showed that the correlation between the results of peer-review grant assessments and the actual impact of the projects in terms of citations is weak (e.g., Doyle et al., 2015; Fang et al., 2016; Van den Besselaar & Sandström, 2015). When it comes to scientific publications, Heesen and Bright (2020) argue that peer review has a high epistemic cost as it is time-consuming while they claim that its benefits are questionable. As a response to such limitations, different types of lotteries are proposed and used as alternatives to traditional grant peer-review (e.g., Adam, 2019). The lottery approach can reduce biases and the workload of the reviewers. Depending on the views on justice and fairness arguments for and against the randomness in decision making can be made. The randomness of the lottery approach might be unsettling as decisions about scientific funding would not be merit based. On the other hand, the machine learning approach is based on citation data from previous projects. This should allow the algorithms to identify the proposals with the highest chance to reach a high number of citations, which is far less arbitrary than a lottery. Another subtlety about the lottery approach is the argument that it should be applied once all proposals passed a certain quality threshold (cf. Adam, 2019). Still, the question remains how the projects are pre-screened in order to reach this category. An algorithmic grant review could be used as a pre-screening method also in this case. It is also questionable how randomly assigned funding would influence the motivation of researchers. For instance, it is possible that this would in a long run demotivate scientists and decrease the quality of project proposals. Finally, the input values of the machine learning model do not include biases of individual reviewers.^3^

Scientific laboratories in many disciplines have grown in size and complexity. In contemporary experimental physics, laboratories often accommodate hundreds of employees and collaborators (e.g., Boisot et al., 2011). The question of whether the initial benefits of an increasing size of research institutions pay off has been thoroughly studied (Carillo et al., 2013; Katz, 1982; Von Tunzelmann et al., 2003). Moreover, various measures of project efficiency in operational and management studies have been developed (e.g., Halkos & Nickolaos, 2007). Measurement of efficiency in industrial economics typically focuses on productivity, i.e., on the actual goal of industrial operations. In science, however, the goal is the production of knowledge.

One can use multiple techniques such as computer simulations, models in decision theory, etc., in order to assess the epistemic efficiency of a group. For instance, prominent approaches for investigating epistemic efficiencies from the perspective of philosophy of science are based on computer simulations (Kelly & Mayo-Wilson, 2010; Zollman, 2007, 2010) and logical models (Baltag et al., 2013).

From the perspective of data science, techniques vary depending on the research goal. For instance, Mutz et al. (2017) applied stochastic frontier analysis (SFA) to scientific projects conducted in Austria in order to measure efficiencies across different scientific fields. The idea of this analysis was to apply SFA to various fields at the same time. Yet, numerical data strongly varies between disciplines. Thus, in the case of our study, we exclusively focused on HEP because of the numerical expressivity of both project information – the number of teams, researchers, and duration of the project, and the outreach of the results. It is important to note that, in the case of Fermilab, researchers were proposing their duration because access to the expensive equipment was scarce. Moreover, the consensus about the results in HEP is relatively quickly adopted, thus reliable due to the underlying structure of the inductive process in the field (Perović & Sikimić, 2019; Schulte, 2000). Still, including more parameters of different kinds would most likely increase the accuracy of the study. We used the data that was available to us.

The predictive analysis assesses the efficiency of a project proposal given the following information about it: the number of proposing authors, the number of teams, and the expected duration of the project. DEA, on the other hand, was used for finding efficient projects and for testing the (input-based) predictive analysis. Finally, our research should serve as a stepping stone towards the optimization of other disciplines with less regular behavior, which has to be tested separately.

## Method

The goal of our research is the pursuit of data-driven optimization techniques of scientific inquiry. Different disciplines require different approaches when it comes to optimization. For instance, in biology, the number of citations is not necessarily appropriate for the estimation of the project’s efficiency, because of the observer bias, problems with the numerical interpretation of the results in some subfields, and ubiquitous non-replicability of results (Pusztai et al., 2013). However, HEP is suitable for a data-driven analysis that uses weighted citations as output due to the unanimity of the results in the field. Using inductive analysis Schulte (2000) concluded the same - that the consensus in HEP is reliable and stable. We analyzed the projects that were run in Fermilab between 1974 and 2003. The more recent experiments are excluded from the analysis because their impact cannot be estimated yet. Moreover, we excluded calibrations of instruments and linked experiments. Having the above-mentioned in mind, we chose Fermilab as one of the most successful laboratories in HEP with a detailed project record.

**Fig. 1:**
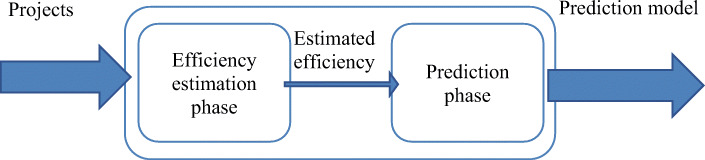
Method for project efficiency estimation and prediction

The proposed method (see Fig. 1) uses two generic phases. The first phase is efficiency estimation. To estimate efficiency, one needs a dataset with two types of columns. Namely, input and output attributes. The first type of columns stands for input attributes, which present project information known at the project application phase. The output attributes are known once the project is finished. The result of this phase is a vector of estimated efficiencies. One can use DEA, SFA, or any other quantitative or qualitative model.

As inputs, we used the number of proposing authors, i.e., the core researchers on a research project, the number of research teams, and the project duration. The supporting staff and engineers working on the experiments are not included as their role across experiments is quite stable and their roles quite remote from the core data production and analysis. Furthermore, they were not included in the internal and external lists of researchers at the time of the experiments. The number of researchers indicated as proposing authors are a reliable indication of the overall team size and the number of research teams into which the researchers are divided. Therefore, the number of input attributes is three. The outputs are the number of citations per paper divided into six weighted categories. The output attributes were the number of famous papers (papers with 250+ citations), the number of very well-known papers (papers with between 100 and 249 citations), the number of well-known papers (papers with between 50 and 99 citations), the number of known papers (papers with between 10 and 49 citations), the number of less-known papers (papers with between 1 and 9 citations), and the number of unknown papers (papers with 0 citations).

The second phase presents the process of creating a predictive model for the estimation of efficiency. This phase takes a dataset containing the project input attributes (the same as for the efficiency estimation) and one or more attributes for project efficiency (the output from the efficiency estimation phase). This phase can be either treated as a classification problem, where the model creates a binary output (the project is efficient or not), or as a regression problem, where the exact value (i.e., percentage) of efficiency is predicted. In this phase, one can use any data mining, machine learning, or expert model. We choose to treat the prediction of efficiency scores as a regression problem because 1) a possible class imbalance problem (few projects are efficient), and 2) the sensitivity of the efficiency estimation phase, i.e., in the case of a project whose efficiency is close to 100%, but still less than 100%, a classification model would not provide the end-user with an adequate result. However, any arbitrary efficiency threshold can be used instead of 100%. This would reduce the class imbalance problem as more projects would be considered as highly efficient. The problem of sensitivity would remain, and the problem of arbitrary selected efficiency threshold could raise additional concerns about potential biases in the interpretation of the decision-making process (similar to the ones in (Ding et al., 2021)).

This setup allows us to obtain an efficiency score for a new project. If we would like to assess the efficiency one would have to add a new project to the dataset and repeat the efficiency estimation procedure. This may be time-consuming, and additionally, we might want to see whether a project will be efficient or not before it ends (when outputs are unavailable). In that case, one needs to use a function fitting method to assess the efficiency function and provide it as a tool for efficiency estimation.

### Data envelopment analysis

To explain the idea of DEA, we provide a motivating example. The idea of the efficiency estimation is that decision making units (DMUs) with the same level of inputs can be efficient even if they produce different outcomes. Imagine that we have two projects that have the same values for input attributes. For example, let the outcomes of interest be the number of papers and the number of completed Ph.D. dissertations. Let the first project have ten papers published without any completed Ph.D. dissertations, and the second project has three completed Ph.D. dissertations, without any paper published. In other words, both projects had the same starting point, but different outcomes. If we are to estimate the efficiency of both projects, we would say that both are efficient. The first one was efficient regarding publishing, and the second was efficient regarding completed Ph.D. dissertations.

We add the third project that has the same inputs, and five papers and one completed Ph.D. dissertation as outcomes. Compared to the other two projects, this one has a higher value of one outcome attribute and a lower value of the other one. In other words, its outcomes are balanced across outcomes. We cannot say that any of the other two projects utilized the inputs better. In other words, other projects might perform worse if they sought balanced outcomes. Therefore, the third project is efficient as well.

Now, let us add the fourth project. It has the same inputs, but it generated three papers and one completed Ph.D. dissertation. One cannot compare the fourth project with the first two. The first two projects might, or might not, perform better than the fourth one. However, the third project generated two more publications (five compared to three in the fourth project) and the same amount of completed Ph.D. dissertations. Since both had the same inputs, we would say that the third project is efficient, and the fourth one is inefficient. In other words, there exists a project that obtained a better outcome using the same inputs.

This example explains the intuition behind the data envelopment analysis. Data envelopment analysis aims to check if other projects utilize the input resources better. Additionally, for each project, we seek the greatest efficiency score. If there are no other projects that utilize the resources better (either having greater outcomes with the same level of inputs, or reaching the same outcomes with the lower level of inputs) then the project is efficient. If there is at least one project that utilizes the resources better, then we claim that the project is inefficient (and we can say which projects are better than the one we observe). As a result, we can create a Pareto frontier of DMUs that represent a set of projects for which there are no other projects that perform better. This means that the other projects either have worse outcomes (inefficient projects), or that they have a better value for one outcome, but a worse value for the other outcome (either efficient or inefficient projects).

The example assumes that the input attributes are the subject of intervention. In other words, the inputs should be different given the fixed outputs (if the project is inefficient). Therefore, these models are called input-oriented models. This is true in most of the applications. However, sometimes outputs are the subject of intervention given the fixed inputs. If that is the case, one should apply output-oriented models. Another important point is the return to scale. Return to scale is an economic concept implemented into DEA with the idea to have different compensation rates of inputs and outputs. More specifically, an increase in inputs results in a (dis)proportionate increase in the output levels. The most common option is to use a constant return to scale. This means that the compensation between inputs and outputs is constant. For example, if the input values for a unit are all doubled, then the unit must produce twice as many output values. Other options are possible, such as increasing or decreasing the return to scale. However, these models can introduce a lot of variability in the results. More specifically, making projects more efficient (Benicio & de Mello, 2015). We assume a constant return to scale.

Formally, DEA is one of the most popular non-parametric methodologies for efficiency estimation. It is based on the idea of maximization of the weighted sum of output attributes given constraints regarding the relation of input attributes and output attributes (Charnes et al., 2013). This methodology is general and thus it is used in a wide variety of application areas, including banking, tourism, education, sports, etc. (Emrouznejad et al., 2014). As a result of a DEA, one obtains efficiency scores that are expressed as a number between zero and one (or 0% and 100% if percentages are used). An efficiency score equal to one indicates that the DMU is on the Pareto frontier, thus making it efficient. However, if the efficiency score is below one, one can state that there is at least one DMU that better utilizes either the inputs of the resource generation process, the outputs of the resource generation process, or both (Charnes et al., 2013). In other words, this DMU is not on a Pareto frontier, thus it is not deemed as efficient. Instead of evaluating the efficiency of the average DMU, DEA uses similar DMUs that utilize the resources better. The mathematical model that satisfies the above-mentioned intuitions is proposed by Charnes et al. (2013) and is given in:

$$ {\displaystyle \begin{array}{c}\begin{array}{c}\begin{array}{c}\mathit{\max}\ {h}_k=\sum \limits_{r=1}^s{\mu}_r{y}_{rk}\\ {}s.t.\end{array}\\ {}\sum \limits_{i=1}^m{v}_i{x}_{ik}=1\\ {}\sum \limits_{r=1}^s{\mu}_r{y}_{rj}-\sum \limits_{i=1}^m{v}_i{x}_{ij}\le 0,j=1,\dots, n\end{array}\\ {}{\mu}_r\ge \varepsilon, r=1,\dots, s\\ {}{v}_i\ge \varepsilon, ir=1,\dots, m\end{array}} $$

where *μ*_*r*_ represents the weight associated with the output attribute *r* and *v*_*i*_ the weight associated with the input attribute *i*. The value *x*_*ik*_ represents input attribute *i* of DMU *k*, while the value *y*_*rk*_ represents output attribute *r* of DMU *k*. The last two constraints ensure non-negativity of weights using the hyper-parameter *ε* that is often set to a very small value such as 0.0001.

The usefulness of the DEA model comes from the interpretability of the results. Besides telling if the DMU is efficient or not (or more specifically, how efficient the project is), we can derive what inputs should be reduced and/or what outcomes should be increased for the DMU to be efficient. More specifically, one can identify the specific improvement areas with respect to optimal DMUs. This is obtained from the dual formulation of the linear mathematical model.

As its main advantages, one can emphasize that DEA is a linear programming optimization problem, which yields a piecewise linear efficiency frontier for the data at hand (Coelli et al., 2005). This means that the efficiency function that is produced by DEA is complex, having the ability to cope with multiple inputs and outputs. Further, since DEA is a non-parametric method, the end-user does not need to have any knowledge about the efficiency function. Additionally, the interpretation of the produced model is simple. Finally, since this is a linear programming method the solution is optimal for the data at hand. As a downside, one can point out that the model needs to be evaluated for overfitting and that the procedure is sensitive to outliers.

One problem that DEA models are facing is the instability of the results (Cooper et al., 2011). Efficiency scores obtained from DEA are considered relative because the efficiency scores are calculated in relation to other DMUs. This means that the efficiency scores are valid only for the data at hand. If one changes the dataset used (for example, remove DMUs, or add new DMUs) the efficiency scores will differ. In other words, instead of using external efficient measures as a baseline for efficient DMU, DEA uses other DMUs. With a greater number of DMUs, this problem vanishes, therefore, one can utilize a rule-of-thumb recommendation for the size of the dataset (Cooper et al., 2011). This rule indicates that the number of DMUs in a DEA model should be greater than all possible combinations of inputs and outputs. More specifically*, n ≥ max{ms, 3(m + s)},* where *n* is the number of DMUs in the DEA model, *m* is the number of input attributes, and *s* is the number of output attributes. In this research, we have 67 DMUs, three input attributes, and six output attributes. Therefore, our model satisfies the given recommendation regarding the number DMUs (67 > 27). To further downsize the effects of the above-mentioned problem and ensure the stability of the obtained efficiency scores, we created additional constraints for the DEA model. Those constraints are:

$$ {\mu}_k-{\mu}_{k+1}\ge 0,\kern0.5em \forall k,k=1,\dots, s-1 $$

Our intuition was to assign higher weights to the papers with more citations. The proposed set of constraints puts a higher weight on a category of papers with more citations compared to the previous one. This will reduce the effect that high numbers of low-quality papers generate efficient projects. For a project to be efficient, it needs to have papers in a higher category.

### Predictive analysis

Machine learning provides methods of data analysis by building descriptive or predictive models that can be used for decision-making. In other words, it offers a variety of techniques that are used to analyze historical facts in order to make predictions about future events. The relevant models are created by extracting information from selected historical data and using them to predict a trend, state, or value of interest (Murphy, 2012).

The problem of efficiency prediction can be represented as a regression problem, i.e., predicting efficiency scores based on project inputs. We used lasso penalized linear regression and ridge penalized linear regression, both being optimized to select the best value of λ (using inner ten-fold cross-validation) such that the error is minimized (Tibshirani, 1996), neural networks (with optimized hyper-parameters and network structure using inner ten-fold cross-validation) and gradient boosted trees (Friedman, 2001).

To get accurate estimates of our models, and to get meaningful and generalizable patterns, we performed 10-fold cross-validation, which is a model validation technique used to estimate how the model results will generalize in future applications of the predictive model. Cross-validation works as follows. First, the dataset is divided into *k* folds (in this research 10). Then *k*-1 folds (in our case 9) are used for model training (i.e., creating a machine learning model), while the remaining one is used for model performance estimation (this phase is called model testing). The performance of the model is saved and the procedure is redone having a different subset of data for model testing. The procedure is repeated ten times, such that each of the *k* folds is used exactly once for model testing. Performances, measured in root mean squared error (RMSE) and mean absolute error (MAE), are averaged and presented with the standard deviation, yet one should be careful when interpreting the standard deviation since performances are not independent. They are, in fact, used as an indicator of possible overfitting. It is worth noticing that we used the same 67 projects in this phase as in the project efficiency estimation phase.

## Results

In this section, we present the DEA results and analyze them. Also, we show the results of the predictive analyses where we applied several algorithms for estimating efficiencies in HEP. Finally, we draw conclusions about project optimization based on the results of the predictive analysis.

### DEA results

We conducted a DEA on the 67 projects extracted from the Inspire-HEP database. The efficiency scores obtained after the DEA procedure are presented in Table 1. The first column represents Fermilab experiments with the project proposal ID.^4^ The second column represents the efficiency score of each project, while the third column (denoted *Benchmarks*) shows the Fermilab project proposal ID (associated with the weight) if a DMU is inefficient, or the number of DMUs that the efficient DMU mentors. Benchmark projects are the projects that are efficient, and as such utilize their resources better by either needing less inputs to generate the same level of outputs, or generate better output with the same level of input. As such they serve as a benchmark for the other projects most similar concerning the input parameters. The weight that is associated with the project tells us how many times the inefficient project has to reduce its inputs to achieve the same outputs as the benchmark efficient project.

**Table 1:** DEA efficiency scores and benchmarks; the efficient projects are highlighted

*DMU*	*SCORE*	*Benchmarks*	*DMU*	*SCORE*	*Benchmarks*	*DMU*	*SCORE*	*Benchmarks*
*0882*	21.67%	*0770* (0.25)	*0632*	29.20%	*0770* (1.58) *0564* (0.12)	*0365*	72.58%	*0398* (0.62)
*0871*	28.90%	*0770* (0.19) *0428* (0.02) *0398* (2.60)	*0623*	25.57%	*0770* (0.32) *0564* (0.01)	*0358*	15.45%	*0770* (0.25)
*0868*	33.21%	*0770* (0.72) *0564* (0.01)	*0597*	16.73%	*0770* (0.48) *0564* (0.04)	*0357*	20.69%	*0770* (0.37) *0398* (0.00)
*0866*	29.84%	*0770* (1.00) *0428* (0.29)	*0591*	19.13%	*0770* (0.35)	*0350*	52.53%	*0770* (0.44)
*0854*	26.29%	*0770* (0.14) *0398* (0.01)	*0573*	43.55%	*0770* (0.08) *0398* (0.60)	*0345*	19.83%	*0770* (0.02) *0428* (0.23) *0398* (0.18)
*0792*	36.28%	*0770* (0.02) *0398* (0.27)	***0564***	**100.00%**	**19**	*0344*	39.39%	*0770* (0.33)
*0789*	23.62%	*0770* (0.61) *0564* (0.04)	*0546*	53.85%	*0770* (0.62) *0564* (0.27)	*0341*	26.08%	*0398* (0.62)
*0774*	9.56%	*0770* (0.12)	*0536*	19.77%	*0770* (0.19) *0398* (0.06)	*0335*	4.10%	*0770* (0.02) *0398* (0.05)
*0773*	4.20%	*0770* (0.12)	*0515*	20.52%	*0770* (0.58) *0564* (0.01)	*0330*	30.51%	*0770* (0.25)
*0772*	56.01%	*0770* (0.67) *0398* (0.58)	*0505*	72.06%	*0398* (2.62)	*0313*	10.13%	*0770* (0.13)
***0770***	**100.00%**	**58**	*0490*	16.28%	*0770* (0.28) *0564* (0.03)	*0311*	81.00%	*0770* (0.98) *0564* (0.06)
*0769*	30.44%	*0770* (0.61) *0564* (0.02) *0398* (0.14)	*0469*	10.52%	*0770* (0.25)	*0305*	21.92%	*0770* (0.33) *0398* (0.07)
*0761*	39.83%	*0770* (0.14) *0428* (0.76) *0398* (1.27)	*0462*	32.53%	*0770* (0.06) *0564* (0.08)			
*0760*	64.82%	*0770* (0.86) *0428* (2.00) *0398* (0.14)	*0461*	20.80%	*0770* (0.02) *0398* (0.27)			
*0756*	29.66%	*0770* (0.05) *0398* (1.42)	*0448*	59.09%	*0770* (0.50)			
*0747*	7.04%	*0770* (0.02) *0398* (0.27)	*0444*	56.77%	*0770* (0.20) *0564* (0.04) *0398* (1.28)			
*0745*	6.81%	*0398* (0.62)	*0439*	18.22%	*0770* (0.17) *0564* (0.03)			
*0744*	10.33%	*0770* (0.20)	*0436*	3.59%	*0770* (0.04) *0398* (0.01)			
*0743*	7.73%	*0770* (0.19) *0564* (0.01) *0398* (0.27)	***0428***	**100.00%**	**5**			
*0735*	63.66%	*0770* (1.43) *0398* (0.63)	*0400*	18.72%	*0770* (0.52)			
*0733*	18.25%	*0398* (1.00)	***0398***	**100.00%**	**31**			
*0713*	5.14%	*0770* (0.09)	*0397*	10.20%	*0770* (0.17)			
*0711*	6.52%	*0770* (0.09) *0398* (0.36)	*0396*	20.29%	*0770* (0.26)			
*0706*	46.07%	*0770* (2.55) *0564* (0.07)	*0388*	8.46%	*0770* (0.18) *0564* (0.03)			
*0705*	52.73%	*0770* (1.21) *0564* (0.32) *0398* (0.37)	*0382*	19.67%	*0770* (0.06) *0398* (0.33)			
*0704*	34.12%	*0770* (1.00) *0398* (2.00)	*0379*	14.43%	*0770* (0.22)			
*0683*	3.63%	*0770* (0.21) *0564* (0.01)	*0369*	42.82%	*0770* (0.62) *0564* (0.05) *0398* (0.14)			

The efficient projects are bolded.

### Qualitative analysis of the DEA results

We analyzed sixty-seven experiments that were run in Fermilab during the transitional period of its development, while it was still managed as a platform for a number of smaller external teams, rather than as a site for a few long-running experiments (Hoddeson et al., 2008). The reason for excluding calibrations of instruments, linked experiments, and precision measurements is that they are disproportionately cited and their tasks are too specialized. In other words, we only considered substantial experiments probing particle dynamics and properties, whose impact can unambiguously be assessed and compared.

The descriptive statistics of the efficiency scores explain that the average efficiency is 32.13% with a standard deviation of 25.55%. This is an indicator that the efficiency scores have a right tail in their distribution, which is not excessively large. Most of the projects have efficiency scores between 20% and 40% (39 of them), but very high-efficiency scores are represented as well. More specifically, nine projects have an efficiency score of over 80%. By inspection of the lower efficiency experiments, we found that most of the projects did not generate a large number of outcomes in well-known papers or higher categories. Due to the design of the mathematical model where papers in a better category are valued more, their score is justifiably reduced.

The following four projects were efficient with regard to the other projects in the dataset, i.e., they scored 100% using data envelopment analysis. Experiment 0770, *Neutrino Physics at the Tevatron*, was a re-run of 0744 that analyzed di-muon decays, crucial for detecting neutrinos. *Direct detection of short-lived particles from neutrino interactions in nuclear emulsions inside the 15 - ft bubble chamber* (0564), *400-GeV proton interactions in nuclear emulsion* (0428), and *A proposal for a further study of muon nucleon inelastic scattering at Fermilab* (0398) turned out efficient as well. The following four projects with an efficiency score below 5% were particularly inefficient: 0773, 0683, 0436, and 0335.

For projects to be efficient, it is mostly needed to increase the number of papers in the best categories, more specifically famous papers, and very well-known papers by 29% and 35%, respectively, and the number of unknown papers by 15%, while other categories are less important. This behavior is expected due to the design of the additional constraints in the mathematical model.

As we mentioned in the Method section, the usability of DEA analysis is in the ability to explain what is needed for a project to be efficient (Aysolmaz et al., 2021). For the problem at hand, we provided several examples. Project 0773 should increase the number of very well-known papers from one to seven, as well as well-known papers from zero to two. In other words, this project failed to generate enough quality outputs to be considered an efficient one. A similar conclusion applies to projects 0683, 0436, and 0335, where the number of very well-known papers and well-known papers should be one for both categories, while the projects did not result in a single paper in both categories. Although all projects required the same increase, they were different in design. The experiment 0683 (*Photoproduction of high p(T) jets*) lasted for 4229 days and it collected a group of three teams and 25 researchers, while experiment 0436 (*Determination of the possible di-muon character of the prompt muon flux*) was small by design, and lasted less than a year, had three research teams, and ten researchers. Experiment 0335 (*A search for direct muon production in the forward direction*) was in between these two projects with the project duration of 521 days, five research teams, and five researchers included. The common point associated with all three projects is the lack of any outcome. These projects managed to generate at most three papers, with all the projects having one paper in the category well-known (and the other being in the lower value categories). Another thing in common for them is the referent efficient project. For all of these projects, the one that is to be looked at is experiment 0770, *Neutrino Physics at the Tevatron*. Experiment 0770 managed to generate high-quality outcomes placing other projects in an inefficient position.

### Predictive analysis in HEP

The calculated efficiency scores were used to investigate the performance of machine learning algorithms for the prediction of the project’s success. The obtained performances for several machine learning algorithms are presented in Table 2. The first column shows the learning algorithm. The second and third columns show the performance metrics RMSE and MAE.

**Table 2:** Predictive performances

Algorithm	RMSE	MAE
Lasso linear regression	0.030 ± 0.030	0.123 ± 0.045
Ridge linear regression	0.031 ± 0.026	0.130 ± 0.042
Neural network	0.054 ± 0.035	0.180 ± 0.057
Gradient boosted trees	0.035 ± 0.026	0.125 ± 0.039

As we can see from Table 2, one cannot clearly decide which algorithm performed the best. However, lasso linear regression has an MAE error of 0.123, which means that there is an average absolute error of 12.3%. Based on the RMSE, we can say that the error level is acceptable (lower than 0.05). These results suggest that we can successfully predict the efficiency of a project at a satisfactory level. In order to examine the efficiency, we created a grid of values ranging from the lowest to the highest value (observed in the entire dataset) with 100 equally distributed dots. We further applied the lasso linear regression model for each such created example and obtained their predicted efficiency score. Finally, we presented this data as a heatmap where blue represents higher efficiency scores while red represents lower ones.

**Fig. 2:**
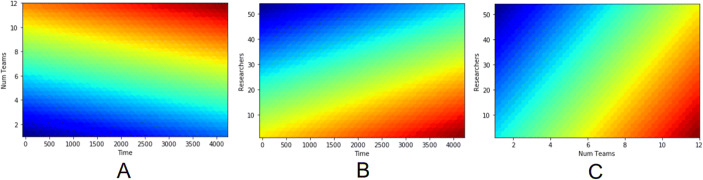
Heatmaps of efficiency. The three input variables time (in days), the number of teams and the number of researchers are plotted against each other. The color indicates the efficiency scale from efficient (blue) to inefficient (red). As we can see, experiments become inefficient if they last long or involve a high number of teams

The diagrams in Fig. 2 visualize the influence of the length of the research project, the number of research teams, and the number of researchers on the efficiency of a project. As we can observe, an important variable is the number of research teams. Projects involving two research teams are most efficient, but projects with six or fewer research teams are efficient as well. The efficiency drops slowly when more teams are getting involved (Fig. 2A, C). Moreover, projects with a large number of researchers should not take too much time to complete (Fig. 2B). Similarly, the higher the number of researchers, the better the efficiency score, as long as the timeframe is short and the number of teams stays small (Fig. 2B, C). To sum up, efficient projects involve two or fewer teams and require less than five hundred days (Fig. 2A, blue).

## Discussion

Our results are valuable for researchers as well as funding agencies. Firstly, during the efficiency estimation phase the projects with optimal structures are identified. The results of this phase can, therefore, contribute to the discussion on efficient group structures and resource allocation in science. Researchers could use our DEA results as indications when it comes to project planning and designing their teams. Secondly, automated grant review based on machine learning would make predictions about efficiency based on the data from the project proposals. Our predictive analysis shows that function fitting methods (e.g., data mining or machine learning algorithms) can be used as one aspect of peer-review to predict the efficiency of the investment with the help of the quantitative data from the proposal.

### Efficient projects in HEP

The results have moderately high accuracy and are insightful when it comes to determining an efficient team size and structure. From the conducted analyses, we can infer two important trends when it comes to structuring research projects in HEP experiments (Table 3). Firstly, the results point out that there is a saturation point (1500 days) after which one should not expect many breakthrough results. In philosophy of science the idea that there might be a saturation point in research is not new. For instance, stopping rules that govern researchers in deciding when to stop data gathering and start with interpreting them were discussed in the literature (Roger, 2012; Steele, 2013). Secondly, all necessary staff should be divided into as few teams as possible to make the collaboration efficient (ideally less than three, but up to six).

**Table 3:** Summary: tendencies of efficient projects

Time	The relationship between time and project efficiency has a saturation point.
Project structure	Efficient projects have a smaller number of teams.

While the first conclusion is very intuitive, the second one might require the adaptation of project plans. Our results indicate that project leaders should search for ways to minimize the number of research teams while getting all the necessary workforce and skills required. This result is supported by the literature (e.g., Perović et al., 2016). A potential explanation of this phenomenon can be that smaller steering groups are easier to coordinate. The important aspect is how well project members interact, i.e., how they are divided, while their absolute number plays a smaller role. This highlights interesting aspects about the communication between researchers, and the way researchers collaborate within teams and with other teams.

### General limitations of the use of predictive algorithms

Our predictive analysis was successful in identifying the efficiency of the projects. There are many advantages of an algorithmic project review: the speed of the review process is increased, researchers save time as they have to do less reviewing, the output of the review process is not random but based on the outputs of previously conducted projects, etc. Moreover, individual biases of reviewers towards the projects proposed by famous institutions or by scientists of a specific gender or race are easier to avoid as such parameters do not enter the algorithmic efficiency analysis (so called fairness through unawareness). However, omitting the *sensitive* attribute does not provide a guarantee that the results are going to be discrimination-free. It is worrying that algorithms have the potential to replicate or even amplify existing biases (Mansoury et al., 2020). An example of an unfair algorithmic prediction were the grades initially assigned to students in the UK in 2020. The UK government implemented an algorithmic grading tool because the final exams could not be held due to the COVID-19 pandemic (Satariano, 2020). Unfortunately, the algorithm took into account both the individual grades and the evaluations of the schools which resulted in downgrading the students from schools in poorer neighborhoods (Satariano, 2020). After a public protest the scores given by the algorithm were dismissed. When considering to apply machine learning in decision making one has to keep track of ethical use of data, their collection, parameter choices, development of algorithms, etc.

### Limitations of algorithmic grant review

We do not suggest that predictions should be the only method in the grant evaluation process. There are both practical and theoretical reasons for this. The main theoretical reason is the fact that scientific success includes creative components but also luck, i.e., predicting high-impact scientific discoveries is a hard task. Many unobservable variables can affect the final project results, such as the motivation of the researchers. These variables algorithms cannot detect. Moreover, as high-impact scientific discoveries are relatively unpredictable, there is a high uncertainty when it comes to predicting a project’s success. This is an inherent problem of the evaluation of project proposals in science.

The practical challenges for the use of machine learning in grant review are related to the variables that are used as inputs and outputs. Which parameters are considered as an epistemic effort and which as an epistemic gain is an open question that also depends on the data availability. We used the input parameters that were made available on the INSPIRE-HEP platform. For instance, in our study, the epistemic gain is measured by the publications ranking, while additional potentially interesting data, such as the number of researchers that made a successful scientific career after the project, were not available. Adding such data would contribute to the accuracy and adequacy of the predictions.

The output variables are not always ideal. Especially in certain scientific disciplines, such as biology, citation patterns are more arbitrary than in others (Perović & Sikimić, 2019). Moreover, natural sciences have very different citation trends than social sciences. For example, in natural sciences researchers cite more recent articles while researchers in social sciences and humanities tend to cite older literature more frequently (Huang & Chang, 2008). The differences in citation patterns can be found even within the same field. For instance, Simko (2015) showed that the number of citations in biology correlates with the number of articles on the same species. Furthermore, citations are not necessarily an indicator of scientific significance. From 32 medical claims that had more than a thousand citations and that Contopoulos-Ioannidis et al. (2008) analyzed, only fourteen were replicated while five were contradicted and in eight more cases it was established that the effect was weaker than initially published.

In addition to the fact that citation patterns are different across disciplines, there are several prominent forms of scientific misconduct related to citation practices. One of them is the occurrence of the so-called citation cartels, i.e., the situations in which researchers that are friends boost each other’s citation counts on purpose (Fister Jr et al., 2016). All this can affect the reliability of the efficiency measures that are based on citations.

The results of our predictive analysis are informative when it comes to HEP and in particular, since the analysis was conducted on the experiments ran between 1974 and 2003, to all HEP experiments performed during the same period irrespective of the home laboratory of a roughly matching structure. It can help with decisions where the difference in the size of the experiments is not drastic. Thus, the results are not applicable to the more recent mega experiments, which are larger to a factor of ten when it comes to the number of researchers. This discrepancy makes them incomparable in an immediate sense. One can find this issue as the *concept drift* problem (Žliobaitė, 2010). However, the problem of concept drift can be overcome by constantly adding data on the existing experiments, including recent mega experiments at CERN, and thus training the presented data-driven analyses. Such an approach would enable us to have a reliable predictive analysis even of contemporary experiments in HEP. Moreover, our twofold methodology can be applied to other disciplines as well.

Still, some projects might not be comparable with other ones in their discipline because they require specific team sizes and techniques, e.g., the Big brain project.^5^ These projects represent outliers and, thus, cannot be compared with other projects. They should be evaluated separately, which has also been the case in the past. Such projects are rare and in practice decisions about their funding are made through a careful evaluation of the associated risks and potential success. When it comes to outliers, machine learning algorithms should not be used because the success of the algorithms depends on the training data.

Another important limitation of an algorithmic grant review system is that its results could be predictable. Researchers could adapt their proposals in a way that satisfies the requirements of the algorithm. However, changing the team structure following guidelines on efficient team communication could also be considered as an objective project advantage. Furthermore, regular updating of the dataset with new projects and incorporation of qualitative assessments would serve as preventive measures against the abuse of the system.

### Solutions: Responsible use of predictive algorithms

The algorithmic grant assessment is a fast and economic option of project assessment that might be applied in the future. However, prior studies and considerations of its benefits and limitations will contribute to its responsible use. When it comes to biases in machine learning, the area of increasing fairness in algorithmic decision-making is well-known and studied, especially in recent years. Therefore, if we define sensitive group(s) we can adjust the proposed framework to include the information about the sensitive attribute, reduce the existing bias, and promote equality. For the DEA one can increase the efficiency score of the unprivileged group (Chen et al., 2020) or mitigate the disparate impact in efficiency scores (Radovanović, Savić, & Delibašić, 2021). Similarly, one can adjust the (machine learning) model learning phase to mitigate unwanted discrimination in decision-making. For example, one can set fairness as a constraint (Zafar et al., 2019) or as a regularization parameter (Radovanović, Petrović,  et al., 2021). The above-mentioned approaches tend to equalize efficiency scores between two groups based on a sensitive attribute, thus helping unprivileged groups (e.g., small and unknown research teams) to reach the desired outcome. These approaches attempt to equalize possible historical and cultural biases that exist in society. In other words, these approaches are associated with affirmative action (Abu-Elyounes, 2020).

One should take into account the inherent features of scientific discoveries when it comes to predicting project efficiency. High-impact scientific discoveries are rare occurrences and, thus, give weak signals in the data. The prediction for these events can be improved in two ways: by adding more projects to the dataset, or by including more details about them. The relevant project features could be the ratio between junior and senior researchers, publishing statistics for each project participant, project expenses, etc. As mentioned in the introduction, adding more parameters would most likely increase the accuracy of the analysis. For instance, the number of graduate students that successfully finish their dissertations as a result of a project, or the placement of early-career researchers, could be considered as relevant output parameters. Moreover, the number and types of prizes that were the result of a project are relevant when evaluating its success. When it comes to enriching the set of input parameters, the number of group leaders on a complex project or the team structure, e.g., hierarchical or egalitarian, would be interesting, since studies show that they influence the efficiency of a team (Sikimić & Herud-Sikimić, 2022). The inclusion of such parameters would all be in line with the proposed methodology for establishing project efficiency, conducting the predictive analysis, and testing its accuracy.

Finally, to improve the predictive accuracy, numerical input values could be combined with qualitative ones. The approach of adding fixed qualitative input values into data envelopment analysis of efficiencies has already been implemented (Kao & Lin, 2011). In the context of grant review, an evaluation of a peer reviewer could be incorporated in the study as a qualitative category that could, for instance, evaluate the cohesion between researchers or their motivation on a Likert-type scale.

The proposed machine learning approach has benefits in comparison to other grant review methods and can be used as one of the parameters in project evaluation. For instance, it can be used as a corrective measure that could lead to financing projects that reviewers have already discarded. Moreover, when quantitative results are combined with qualitative ones, we will get a more accurate overall evaluation. Our two-step methodology of combining techniques for efficiency evaluation with predictive algorithms is flexible and can achieve exactly that: it can be adapted to incorporate a qualitative approach such as peer-review into a machine learning assessment.

## Conclusions

We proposed a two-step method for analyzing the efficiency of experiments in HEP. We employed DEA for the optimization of research in HEP and proposed lasso linear regression as an adequate method for predicting project efficiency. The results of the conducted analyses can be useful both for funding agencies and scientists themselves since they predict project efficiency based on the information available in the project proposal. The results show us relevant trade-offs between different parameters and are, thus, instructive when it comes to structuring new experiments. According to our results, shorter experiments outperform long ones. Projects with fewer research teams are more efficient, while the absolute number of researchers is less important for project efficiency. This method can potentially be employed in practice as a corrective method or one of the steps in grant review. Gradually adding new data to our existing analysis would make the analysis informative with respect to new, large experiments. Finally, our approach can be a useful guideline for further data-driven analyses of scientific projects across different research fields.

## Footnotes

1 Data about the funding of the more recent projects have also become available.

2 The analyzed experiments were conducted between 1974 and 2003 to ensure that the number of citations can be reliably evaluated. We collected the citation count for all projects in October 2020. After this timeframe a significant number of new citations or publications from the projects is not likely. In other words, to ensure a stable citation number, the cut-off criterion for projects to be included in the study was 17 years and older.

3 Still, general biases in science might be mirrored in the citation frequencies of certain contributions.

4 The full list of Fermilab project proposals can be found on the following link: http://ccd.fnal.gov/techpubs/fermilab-reports-proposal.html. Based on the proposal number, one can find more details about the approved and conducted experiments.

5 More information about the Big brain project can be found on the following link: <https://bigbrainproject.org>.
